# Fibrilação Atrial e Sepse em Pacientes Idosos e sua Associação com Mortalidade Intra-hospitalar

**DOI:** 10.36660/abc.20220295

**Published:** 2023-02-23

**Authors:** Michele Ouriques Honorato, Juscelio Trajano de Sousa, Luiz Frederico Bezerra Honorato, Nathalia Watanabe, Gabriela Machado Goulart, Rogério Ruscitto do Prado

**Affiliations:** 1 Hospital Sírio-libanês São Paulo SP Brasil Hospital Sírio-libanês, São Paulo, SP – Brasil; 2 Universidade do Extremo Sul Catarinense Curso de Medicina Criciúma SC Brasil Universidade do Extremo Sul Catarinense Curso de Medicina, Criciúma, SC – Brasil; 3 Hospital Israelita Albert Einstein São Paulo SP Brasil Hospital Israelita Albert Einstein, São Paulo, SP – Brasil

**Keywords:** Arritmias Cardíacas, Fibrilação Atrial, Idoso, Sepse, Hospitalização, Mortalidade Hospitalar

## Abstract

**Fundamento:**

A fibrilação atrial (FA) acomete cerca de 2% a 4% da população mundial. Nos pacientes internados em unidades de terapia intensiva, esta incidência pode chegar em até 23% naqueles com choque séptico. O impacto da FA nos pacientes sépticos se reflete em piores desfechos clínicos e o reconhecimento dos fatores desencadeantes pode ser alvo para estratégias de tratamento e prevenção futuras.

**Objetivos:**

Verificar a relação entre desenvolvimento de FA e mortalidade nos pacientes acima de 80 anos incluídos no protocolo sepse e identificar fatores de risco que contribuam para o desenvolvimento de FA nesta população.

**Métodos:**

Estudo observacional retrospectivo, com revisão de prontuários eletrônicos e inclusão de 895 pacientes com 80 anos ou mais, incluídos no protocolo sepse de um hospital privado de alta complexidade em São Paulo/SP, no período de janeiro de 2018 a dezembro de 2020. Todos os testes foram realizados com nível de significância de 5%.

**Resultados:**

A incidência de FA na amostra foi de 13%. Após análise multivariada por regressão logística múltipla, foi possível demonstrar associação de mortalidade na população estudada, com o escore SOFA ( *odds ratio* [OR] 1,21 [1,09 – 1,35]), valores mais altos de proteína C-reativa (OR 1,04 [1,01 – 1,06]), necessidade de droga vasoativa (OR 2,4 [1,38 – 4,18]), uso de ventilação mecânica (OR 3,49 [1,82 – 6,71]) e principalmente FA (OR 3,7 [2,16 – 6,31).

**Conclusões:**

No paciente grande idoso (80 anos ou mais) com sepse, o desenvolvimento de FA se mostrou como fator de risco independente para mortalidade intra-hospitalar.

## Introdução

A fibrilação atrial (FA) é a arritmia cardíaca sustentada mais comum, acometendo cerca de 2% a 4% da população mundial, o que implica em grande morbimortalidade e altos custos para os serviços de saúde.^1 , 2^ Dentre os pacientes que necessitam de internação hospitalar por qualquer outro motivo, a FA segue como a arritmia cardíaca mais detectada, especialmente em pacientes críticos. O risco de desenvolvimento de FA em pacientes admitidos na unidade de terapia intensiva varia entre 4,5% e 11%, chegando até 23% naqueles com choque séptico.^3 , 4^

Dentre os vários fatores de risco estabelecidos para FA, talvez a idade seja o mais proeminente, e o aumento da longevidade da população deve produzir um crescente número de novos casos,^1 , 2^ o que torna os idosos, principalmente os grandes idosos (aqueles com idade acima de 80 anos), mais suscetíveis aos efeitos deletérios e riscos já conhecidos decorrentes da doença, agravados inclusive por outras comorbidades, frequentes nesta população, e sua potencial fragilidade.

Apesar de já existir uma relação bem estabelecida entre idade e FA,^5^ os mecanismos responsáveis pelo desenvolvimento da arritmia em pacientes críticos não são totalmente compreendidos. Provavelmente decorrem de um remodelamento atrial acelerado em combinação com fatores desencadeantes da arritmogênese, habitualmente encontrados em doentes mais graves,^4^ como inflamação, distúrbios hidroeletrolíticos e medicações pró-arrítmicas, dentre elas vasopressores e inotrópicos.^6^

O impacto da FA nesta população se reflete em piores desfechos clínicos, destacando-se por sua importância o aumento no tempo de internação hospitalar, o que infere inclusive em custos, aumento no tempo de ventilação mecânica e suas consequências, e maior mortalidade.^7 - 10^ Entretanto, a sua importância no ambiente crítico ainda não está totalmente clara, ora funcionando como um determinante da piora do paciente, ora como um marcador de gravidade de doenças de base, podendo ser utilizada inclusive como um fator prognóstico.^3 , 6^

Dentro ainda de várias incertezas existentes no ambiente crítico alusivas à FA, a relação entre a arritmia e sepse, principalmente em idosos, ainda suscita diversas teorias. Apesar de estudos crescentes sobre sepse na última década, poucos trabalhos abordaram sua relação com FA em uma população muito idosa (80 anos ou mais). Estes pacientes talvez sejam os mais beneficiados em se manter em ritmo sinusal ou terem a FA revertida o mais breve possível, visto que são habitualmente mais frágeis e têm menor reserva funcional.

Diante do que foi posto anteriormente, torna-se importante e extremamente relevante um estudo que aborde o tema, trazendo informações adicionais que possam contribuir com a literatura vigente. Baseado nisso, este trabalho tem como objetivo primário verificar a associação de mortalidade intra-hopitalar com o desenvolvimento de FA nos pacientes com sepse e, entre os objetivos secundários, identificar potenciais fatores de risco que contribuam para o desenvolvimento de FA nesta população e comparar o tempo de internação entre os pacientes que desenvolveram FA e aqueles que permaneceram em ritmo sinusal.

## Métodos

Trata-se de um estudo observacional retrospectivo, com coleta de dados secundários a partir de prontuários eletrônicos revisados de pacientes com 80 anos ou mais, incluídos no protocolo sepse de um hospital privado de alta complexidade em São Paulo/SP, no período de janeiro de 2018 a dezembro de 2020. O Trabalho foi aprovado pelo Comitê de Ética em Pesquisa do Hospital Sírio Libanês, Protocolo CAAE 47665721.9.0000.546.

Para definir FA, foram utilizados dados do ritmo cardíaco descritos nos prontuários e, quando disponíveis, registros de eletrocardiograma de 12 derivações anexados.

O protocolo sepse utilizado pelo serviço, o qual foi baseado na definição da diretriz da *Surviving Sepsis Campaign* (SEPSIS-3) de 2016,^11^ consistia em foco infeccioso possível ou provável associado a dois dos seguintes marcadores de síndrome da resposta inflamatória sistêmica: frequência cardíaca > 90 bpm, temperatura corporal > 38°C ou < 36°C, frequência respiratória > 20 irpm, leucócitos > 12.000/mm^3^ ou < 4000/mm^3^; ou pelo menos um marcador de disfunção orgânica, caracterizados por: lactato > 22 mg/dL, creatinina > 2,0 mg/dL, bilirrubina > 2,0 mg/dL, índice de normalização internacional > 1,5, tempo de tromboplastina ativada > 60 segundos ou plaquetas < 100.000/mm^3^.

Estavam aptos à inclusão 1339 prontuários e foram excluídos 444 que se apresentavam em arritmia à admissão ou eram portadores de marca-passo cardíaco. Restaram 895 pacientes admitidos em ritmo sinusal, dentre os quais, 14,9% já tinham apresentado FA paroxística previamente ( Figura 1 ).

**Figura Central:**
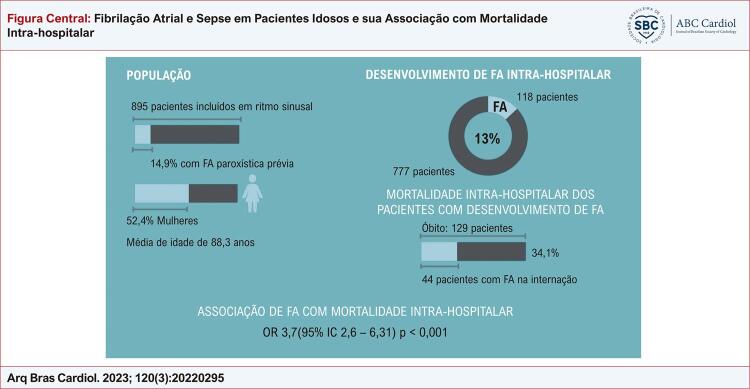
: Fibrilação Atrial e Sepse em Pacientes Idosos e sua Associação com Mortalidade Intra-hospitalar

Resumo dos principais resultados encontrados. FA: fibrilação atrial; IC: intervalo de confiança; OR: odds ratio.

As seguintes variáveis foram analisadas: sexo, idade, índice de massa corporal, tempo de internação hospitalar, mortalidade intra-hospitalar, comorbidades associadas (hipertensão arterial sistêmica, insuficiência cardíaca, diabetes mellitus, acidente vascular cerebral, doença arterial coronariana crônica, FA prévia, doença renal crônica dialítica, obesidade ou outras — demais comorbidades não citadas), uso de antiarrítmico prévio (amiodarona, betabloqueador e outros — bloqueador de canal de cálcio, digoxina, propafenona, etc.), dados ecocardiográficos como tamanho do átrio esquerdo e fração de ejeção do ventrículo esquerdo (FEVE), foco da sepse (pulmonar, urinário, abdominal, cutâneo e outros — cateter, sistema nervoso central, etc.), escore SOFA (caracterizado pela soma de valores atribuídos de 0 a 4 a cada uma das seguintes variáveis: relação PaO_2_/FiO_2_, contagem plaquetária, valores de bilirrubinas totais, pressão arterial média, escala de coma de Glasgow, níveis de creatinina ou quantidade do débito urinário),^11^ valor de proteína C-reativa (PCR) na admissão, uso de drogas vasoativas (noradrenalina, vasopressina e/ou dobutamina) e necessidade de ventilação mecânica.

A definição de insuficiência cardíaca baseou-se nas comorbidades prévias descritas em prontuários e/ou nos exames de ecocardiografia prévios do paciente demonstrando FE < 40% (Simpsom ou Teicholz), valor que, de acordo com a Diretriz Brasileira de Insuficiência Cardíaca Crônica e Aguda, a caracteriza como insuficiência cardíaca de fração de ejeção reduzida.^12^ Átrio esquerdo aumentado foi definido como a medida linear > 40 mm (valor de referência entre 28 e 40 mm). Resultados de PCR acima de 1 mg/dL, obtido pelo método imunoturbidimetria ultrassensível, devem ser interpretados como indicativos de possível processo infeccioso ou inflamatório.

### Análise estatística

As características qualitativas foram descritas com o uso de frequências absolutas e relativas, baseadas no desenvolvimento de FA e foi verificada a associação das características com os grupos com uso de testes qui-quadrado ou testes exatos (teste exato de Fisher ou teste da razão de verossimilhanças).^13^ As características quantitativas foram descritas, segundo o desenvolvimento de FA, com uso de média e desvio padrão quando a distribuição dos dados foi normal ou mediana e quartis quando a distribuição dos dados não apresentou normalidade, sendo avaliada a normalidade com uso do teste Kolmogorov-Smirnov, e comparadas com uso do teste t-Student não pareado ou testes Mann-Whitney, respectivamente.

Foram estimados os *odds ratios* (OR) não ajustados para cada característica avaliada para o desfecho com uso de regressão logística bivariada e foi estimado o modelo de regressão logística múltipla, selecionando-se as variáveis que nos testes bivariados apresentaram níveis de significância inferiores a 0,20 (p < 0,20), sendo todas as variáveis inseridas no modelo, mantidas no modelo final ( *full model* ).

As análises foram realizadas com uso do *software* IBM-SPSS para Windows versão 22.0 e tabuladas com uso do *software* Microsoft-Excel 2010; os testes foram realizados com nível de significância de 5%.

## Resultados

A média de idade da população estudada foi de 88,3 anos (± 5,3), sendo 52,4% mulheres. Durante a internação 118 pacientes evoluíram com FA, representando aproximadamente 13% do total de indivíduos acompanhados retrospectivamente ( Figura 1 ).

Como esperado, os pacientes que desenvolveram FA tinham uma frequência maior de insuficiência cardíaca e FA prévia em seu histórico, bem como um átrio esquerdo aumentado e FEVE reduzida no ecocardiograma.

Foi observado, entre os pacientes que evoluíram com FA, valores maiores do escore SOFA e níveis mais elevados de PCR.

Estes pacientes com arritmia permaneceram mais tempo internados, necessitaram mais frequentemente de drogas vasoativas e ventilação mecânica. Todos os dados acima encontram-se detalhados na Tabela 1 .

**Tabela 1:** – Descrição das características avaliadas segundo desenvolvimento de fibrilação atrial e resultado dos testes estatísticos

Variável	FA na internação		Total (N = 895)	p

	Não (N = 777)	Sim (N = 118)		
**Idade, média ± DP**	88,2 ± 5,2	89,1 ± 5,6	88,3 ± 5,3	0,080**
**Sexo, n (%)**				0,818
Masculino	371 (47,7)	55 (46,6)	426 (47,6)	
Feminino	406 (52,3)	63 (53,4)	469 (52,4)	
**IMC (Admissão), média ± DP**	25,3 ± 4,9	25,1 ± 4,8	25,3 ± 4,9	0,634**
**Tempo de permanência (em dias), mediana (IIQ)**	10 (7; 16)	13,5 (8; 25,3)	10 (7; 17)	<0,001£
**HAS, n (%)**				0,145
Não	325 (41,8)	41 (34,7)	366 (40,9)	
Sim	452 (58,2)	77 (65,3)	529 (59,1)	
**DM, n (%)**				0,420
Não	523 (67,3)	75 (63,6)	598 (66,8)	
Sim	254 (32,7)	43 (36,4)	297 (33,2)	
**Insuficiência cardíaca, n (%)**				0,002
Não	634 (81,6)	82 (69,5)	716 (80)	
Sim	143 (18,4)	36 (30,5)	179 (20)	
**AVC, n (%)**				0,321
Não	665 (85,6)	105 (89)	770 (86)	
Sim	112 (14,4)	13 (11)	125 (14)	
**DAC crônica, n (%)**				0,647
Não	588 (75,7)	87 (73,7)	675 (75,4)	
Sim	189 (24,3)	31 (26,3)	220 (24,6)	
**FA, n (%)**				<0,001
Não	688 (88,5)	74 (62,7)	762 (85,1)	
Sim	89 (11,5)	44 (37,3)	133 (14,9)	
**DRC não dialítica, n (%)**				0,739*
Não	760 (97,8)	115 (97,5)	875 (97,8)	
Sim	17 (2,2)	3 (2,5)	20 (2,2)	
**Obesidade, n (%)**				0,969
Não	515 (66,3)	78 (66,1)	593 (66,3)	
Sim	262 (33,7)	40 (33,9)	302 (33,7)	
**Outras, n (%)** ^**a**^				0,269*
Não	24 (3,1)	6 (5,1)	30 (3,4)	
Sim	753 (96,9)	112 (94,9)	865 (96,6)	
**Foco da sepse, n (%)**				0,886#
Pulmonar	436 (56,1)	71 (60,2)	507 (56,6)	
Urinária	181 (23,3)	23 (19,5)	204 (22,8)	
Abdominal	90 (11,6)	13 (11)	103 (11,5)	
Cutânea	23 (3)	3 (2,5)	26 (2,9)	
Outras ^b^	47 (6)	8 (6,8)	55 (6,1)	
Antiarrítmico, n (%)				0,053
Não	493 (63,4)	63 (53,4)	556 (62,1)	
Amiodarona	91 (11,7)	24 (20,3)	115 (12,8)	
Betabloqueador	149 (19,2)	24 (20,3)	173 (19,3)	
Outros ^c^	44 (5,7)	7 (5,9)	51 (5,7)	
**AE (> 40 mm), n (%)&**				0,021
Normal	308 (46,7)	38 (34,9)	346 (45,1)	
Aumentado	351 (53,3)	71 (65,1)	422 (54,9)	
**FE (< 40% ), n (%)&**				0,040
Normal	621 (94,2)	97 (89)	718 (93,5)	
Diminuída	38 (5,8)	12 (11)	50 (6,5)	
**Escore SOFA na admissão, média ± DP**	3,2 ± 2,1	3,8 ± 2,6	3,3 ± 2,2	0,004**
**PCR, mediana (IIQ)**	4,6 (1,4; 10,7)	7,6 (2,2; 14)	4,8 (1,4; 11,2)	0,027£
**DVA, n (%)**				<0,001
Não	615 (79,2)	65 (55,1)	680 (76)	
Sim	162 (20,8)	53 (44,9)	215 (24)	
**VM, n (%)**				<0,001
Não	729 (93,8)	90 (76,3)	819 (91,5)	
Sim	48 (6,2)	28 (23,7)	76 (8,5)	

Teste qui-quadrado; * Teste exato de Fisher; # Teste da razão de verossimilhanças; ** Teste t-Student; £ Teste Mann-Whitney; & Nem todos possuem a informação. AE: átrio esquerdo; AVC: acidente vascular cerebral; DAC: doença arterial coronariana; DM: diabetes mellitus; DP: desvio padrão; DRC: doença renal crônica; DVA: droga vasoativa; FA: fibrilação atrial; FE: fração de ejeção; HAS: hipertensão arterial sistêmica; IIQ: intervalo interquartil; IMC: índice de massa corpórea; PCR: proteína C-reativa; SOFA: Sequential Organ Faiulure Assessment; VM: ventilação mecânica. ^*a*^ Demais comorbidades não citadas. ^*b*^ Cateter, sistema nervoso central, etc. ^*c*^ Bloqueador de canal de cálcio, digoxina, propafenona, etc.

A taxa de mortalidade intra-hospitalar entre os pacientes que desenvolveram FA foi de 34,1% ( Figura 1 ). Estes pacientes permaneceram mais tempo internados, e os seguintes fatores contribuíram para o desfecho desfavorável, segundo análise estatística: comorbidades como insuficiência cardíaca prévia, o foco inicial da sepse, escore SOFA mais alto, valores mais elevados de PCR, necessidade de droga vasoativa, necessidade de ventilação mecânica e como suspeitado, o desenvolvimento de FA. Todos os dados acima encontram-se detalhados na Tabela 2 .

**Tabela 2:** – Descrição dos desfechos dos pacientes segundo as características avaliadas e resultado das análises não ajustadas

Variável	Status vital do paciente na alta		OR	IC (95%)		p
	Vivo (N = 766)	Morto (N = 129)		Inferior	Superior	
**Idade, média ± DP**	88,3 ± 5,2	88,4 ± 5,6	1,00	0,97	1,04	0,815**
**Sexo, n (%)**						0,909
Masculino	364 (85,4)	62 (14,6)	1,00			
Feminino	402 (85,7)	67 (14,3)	0,98	0,67	1,42	
**IMC (Admissão), média ± DP**	25,4 ± 4,8	24,8 ± 5,4	0,98	0,94	1,02	0,224**
**Tempo de permanência (em dias), mediana (IIQ)**	10 (7; 16)	13 (5; 29)	1,01	1,01	1,02	**0,027£**
**HAS, n (%)**						0,664
Não	311 (85)	55 (15)	1,00			
Sim	455 (86)	74 (14)	0,92	0,63	1,34	
**DM, n (%)**						0,870
Não	511 (85,5)	87 (14,5)	1,00			
Sim	255 (85,9)	42 (14,1)	0,97	0,65	1,44	
**Insuficiência cardíaca, n (%)**						**0,008**
Não	624 (87,2)	92 (12,8)	1,00			
Sim	142 (79,3)	37 (20,7)	1,77	1,16	2,70	
**AVC, n (%)**						0,580
Não	657 (85,3)	113 (14,7)	1,00			
Sim	109 (87,2)	16 (12,8)	0,85	0,49	1,50	
**DAC crônica, n (%)**						0,298
Não	573 (84,9)	102 (15,1)	1,00			
Sim	193 (87,7)	27 (12,3)	0,79	0,50	1,24	
**FA, n (%)**						0,964
Não	652 (85,6)	110 (14,4)	1,00			
Sim	114 (85,7)	19 (14,3)	0,99	0,58	1,67	
**DRC não dialítica, n (%)**						>0,999*
Não	749 (85,6)	126 (14,4)	1,00			
Sim	17 (85)	3 (15)	1,05	0,30	3,63	
**Obesidade, n (%)**						0,915
Não	507 (85,5)	86 (14,5)	1,00			
Sim	259 (85,8)	43 (14,2)	0,98	0,66	1,45	
**Outras, n (%)** ^**a**^						0,295*
Não	28 (93,3)	2 (6,7)	1,00			
Sim	738 (85,3)	127 (14,7)	2,41	0,57	10,24	
**Foco da sepse, n (%)**						**0,008#**
Pulmonar	442 (87,2)	65 (12,8)	1,00			
Urinária	182 (89,2)	22 (10,8)	0,82	0,49	1,37	
Abdominal	78 (75,7)	25 (24,3)	2,18	1,30	3,67	
Cutânea	22 (84,6)	4 (15,4)	1,24	0,41	3,70	
Outras ^b^	42 (76,4)	13 (23,6)	2,11	1,07	4,13	
**Antiarrítmico, n (%)**						0,889
Não	479 (86,2)	77 (13,8)	1,00			
Amiodarona	96 (83,5)	19 (16,5)	1,23	0,71	2,13	
Betabloqueador	147 (85)	26 (15)	1,10	0,68	1,78	
Outros ^c^	44 (86,3)	7 (13,7)	0,99	0,43	2,28	
**AE (> 40 mm), n (%)**						0,765
Normal	295 (85,3)	51 (14,7)	1,00			
Aumentado	363 (86)	59 (14)	0,94	0,63	1,41	
**FE (< 40%), n (%)**						**0,015**
Normal	621 (86,5)	97 (13,5)	1,00			
Diminuída	37 (74)	13 (26)	2,25	1,15	4,38	
**Escore SOFA na admissão, média ± DP**	3 ± 1,9	4,8 ± 2,9	1,40	1,29	1,52	<0,001**
**PCR, mediana (IIQ)**	4,4 (1,3; 10,6)	8,1 (2,6; 17,9)	1,05	1,03	1,07	**<0,001£**
**DVA, n (%)**						**<0,001**
Não	626 (92,1)	54 (7,9)	1,00			
Sim	140 (65,1)	75 (34,9)	6,21	4,18	9,22	
**VM, n (%)**						**<0,001**
Não	731 (89,3)	88 (10,7)	1,00			
Sim	35 (46,1)	41 (53,9)	9,73	5,89	16,08	
**FA na internação, n (%)**						**<0,001**
Não	692 (89,1)	85 (10,9)	1,00			
Sim	74 (62,7)	44 (37,3)	4,84	3,13	7,49	

Teste qui-quadrado; * Teste exato de Fisher; # Teste da razão de verossimilhanças; ** Teste t-Student; £ Teste Mann-Whitney. AE: átrio esquerdo; AVC: acidente vascular cerebral; DAC: doença arterial coronariana; DM: diabetes mellitus; DP: desvio padrão; DRC: doença renal crônica; DVA: droga vasoativa; FA: fibrilação atrial; FE: fração de ejeção; HAS: hipertensão arterial sistêmica; IC: intervalo de confiança; IIQ: intervalo interquartil; IMC: índice de massa corpórea; OR: odds ratio; PCR: proteína C-reativa; SOFA: Sequential Organ Faiulure Assessment; VM: ventilação mecânica. ^*a*^ Demais comorbidades não citadas. ^*b*^ Cateter, sistema nervoso central, etc. ^*c*^ Bloqueador de canal de cálcio, digoxina, propafenona, etc.

Após análise multivariada por regressão logística múltipla, foi possível demonstrar associação de mortalidade na população estudada com o escore SOFA, valores mais altos de PCR, necessidade de droga vasoativa, uso de ventilação mecânica e principalmente FA ( Tabela 3 ) ( Figura 1 ).

**Tabela 3:** – Resultado do modelo ajustado para explicar o óbito nos pacientes do protocolo sepse com 80 anos ou mais

Variável	OR	IC (95%)		p

		Inferior	Superior	
**Insuficiência cardíaca**	1,60	0,91	2,81	0,104
**Foco da sepse**				
Pulmonar	1,00			
Urinária	1,26	0,68	2,34	0,457
Abdominal	1,50	0,74	3,05	0,263
Cutânea	0,78	0,16	3,75	0,752
Outras ^a^	1,37	0,56	3,35	0,494
**FE (< 40%) - ECO**	1,09	0,47	2,55	0,844
**Escore SOFA na admissão**	1,21	1,09	1,35	**<0,001**
**PCR**	1,04	1,01	1,06	**0,007**
**DVA**	2,40	1,38	4,18	**0,002**
**VM, n (%)**	3,49	1,82	6,71	**<0,001**
**FA na internação, n (%)**	3,70	2,16	6,31	**<0,001**

*Regressão logística múltipla (full model). DVA: droga vasoativa; FA: fibrilação atrial; FE: fração de ejeção; IC: intervalo de confiança; OR: odds ratio; PCR: proteína C-reativa; SOFA Sequential Organ Faiulure Assessment; VM: ventilação mecânica. ^*a*^ Cateter, sistema nervoso central, etc.*

## Discussão

A incidência global de FA nova ou recorrente encontrada em nossa população, durante a internação, foi de 13,2%. O que se percebe na literatura é uma grande variabilidade de resultados, por exemplo, em uma metanálise conduzida por Kuipers et al.,^14^ a incidência de FA em pacientes com sepse, sepse grave e choque séptico foi respectivamente de 8%, 10% e 23%.^15 , 16^ No estudo de Walkey et al.,^17^ incluindo mais de 40 mil pacientes, mas apenas com sepse grave (classificação antiga), foi encontrada uma incidência de 5,9% de FA nova. Já em Meierhenrich et al.,^9^ foram selecionados apenas pacientes com choque séptico e foi encontrada uma incidência de 46% de FA nova, 10 vezes mais do que aqueles pacientes com sepse sem evolução para choque. Entretanto, mais recentemente, a metanálise conduzida por Corica et al.,^18^ demonstrou prevalência de FA nova em pacientes sépticos de 13,5%, semelhante à do presente estudo. Essa variabilidade pode ser explicada pelos inúmeros critérios de inclusão e diferentes populações abordadas. Este estudo, por sua vez, representa uma parcela muito específica da população idosa cuja incidência de FA é maior, além de não diferenciar aqueles com sepse e choque séptico.

Dentre os fatores de risco que contribuíram para o desenvolvimento de FA durante a internação, destacaram-se o histórico prévio de insuficiência cardíaca e FA e achados ecocardiográficos que caracterizavam aumento do átrio esquerdo e redução da FEVE. Diversos trabalhos que abordaram o tema também tiveram impressões semelhantes,^4 , 6 , 15^ mas há controvérsias. Salman et al.,^19^ analisando uma coorte prospectiva, não demonstraram relação entre a apresentação de FA e o tamanho do átrio esquerdo, apesar de associar a queda na FEVE com maior chance de evolução para arritmia. Já Shaver et al.,^15^ em direção oposta, conseguiram demonstrar associação com o tamanho do átrio esquerdo, mas não com a FEVE. Apesar de não ser alvo deste trabalho, ensaios laboratoriais com o peptídeo natriurético cerebral o colocam em posição de marcador independente no desenvolvimento de FA, como cita Augusto et al.,^20^ Isso mais uma vez corrobora que a insuficiência cardíaca é um preditor para o surgimento da arritmia tanto ambulatorialmente como no ambiente crítico.^21 - 23^

Também foi observado neste trabalho, entre os pacientes que evoluíram com FA, valores maiores do escore SOFA e níveis mais elevados de PCR. Hoje, o papel inflamatório gerado pela sepse tem significativa importância no desenvolvimento e manutenção da FA. Steinber et al.,^24^ destacam que o processo inflamatório predispõe ao estresse oxidativo, apoptose e fibrose, gerando um substrato importante para desencadear a arritmia. Outro agravante nesse contexto é o ambiente pró-trombótico produzido pela inflamação, capaz de induzir disfunção endotelial, ativação plaquetária e a cascata de coagulação. Ambos os efeitos podem ser responsáveis não só pelo início e manutenção da FA, mas também piorar os desfechos trombóticos associados à arritmia.^24^ Estes achados foram reforçados por Harada et al.,^25^ que também associaram maiores valores de PCR e escore SOFA ao desenvolvimento de FA, inclusive a mortalidade, após análise ajustada. Launey et al.,^23^ também encontraram valores maiores de escore de SOFA em pacientes que evoluíram com FA. Meierhenrich et al.,^9^ evidenciaram que pacientes que desenvolveram FA, sépticos ou não, apresentaram níveis mais altos de PCR antes do evento arrítmico. Chung et al.,^26^ também demonstraram associação entre valores elevados de PCR e ocorrência e manutenção da FA. Nestes casos, o PCR elevado aponta para um estado inflamatório que promove o desenvolvimento ou a persistência de FA.

Inicialmente, sepse de foco abdominal ou outro (não urinário, pulmonar ou cutâneo) foi associado a maior mortalidade, entretanto, na análise multivariada, não foi encontrada diferença estatística. Apesar de existirem dados que apontem maior incidência de FA com infecções do trato respiratório e foco urinário,^10 , 14 , 16 , 23^ em nosso estudo, não foi possível determinar a relação entre os sítios de infecção e a incidência de FA.

Além de permanecerem mais tempo internados neste estudo, pacientes que desenvolveram FA também necessitaram mais frequentemente de droga vasoativa e ventilação mecânica. O uso de ventilação mecânica já foi evidenciado como fator de risco para FA em diversos estudos,^4 , 7 , 21^ mas ainda não é um consenso.^15^ Em pacientes com sepse que evoluem para choque, a necessidade de droga vasoativa se torna imperativa pela falha na compensação entre a demanda e a oferta de oxigênio aos tecidos, portanto, por si só, já é um fator de pior prognóstico. Esta instabilidade pode resultar em FA assim como a própria arritmia gera necessidade de doses mais altas de vasopressores. Esta relação já foi reproduzida em alguns estudos.^4 , 15 , 22^

Diversos trabalhos anteriores falharam em encontrar uma associação direta de FA nova ou recorrente com o desfecho de morte durante a internação, mas os resultados encontrados aqui, sugerem que o desenvolvimento de FA em pacientes sépticos, nesta faixa etária, tem forte impacto sobre a mortalidade intra-hospitalar. Tal achado vai de encontro a outros trabalhos recentes, incluindo metanálises de pacientes críticos, com sepse e/ou choque séptico que desenvolveram FA nova, os quais exibiram resultados semelhantes.^8 , 10 , 13 , 14^

Houve sempre uma grande discussão se a FA tinha um papel a desempenhar na evolução ou desfecho da sepse, ou simplesmente refletia a severidade da doença, como um marcador de gravidade. Como outros trabalhos referidos anteriormente aqui, a FA representou sim uma disfunção orgânica que implicou em piora do desfecho clínico. Diante do que foi exposto, a abordagem adequada da arritmia deve ser elevada a outro patamar de importância no contexto da evolução deste grupo de pacientes, sendo necessário o desenvolvimento de estratégias adequadas de prevenção e tratamento, visando redução de danos.^15^ Especificamente sobre este tópico, já existem resultados que mostraram que a falha na manutenção do ritmo sinusal em pacientes com sepse foi associada a piores taxas de mortalidade em comparação com aqueles pacientes que obtiveram sucesso na reversão da FA.^9 , 16^

Este estudo possui algumas limitações, como a definição de FA, que foi baseada em registros clínicos em prontuários e, quando disponíveis, eletrocardiograma de 12 derivações. Isto deixa espaço para possíveis episódios de FA paroxística não diagnosticada ou registrada pelo médico responsável. Também foram incluídos pacientes com FA recorrente, mas isto não se mostrou como viés de amostragem com implicação nos desfechos. Tal fato já tinha sido abordado em trabalhos realizados por Arrigo et al.,^3^ e Shaver et al.,^15^ que evidenciaram maiores taxas de mortalidade em pacientes com FA nova, provavelmente por menor tolerância às alterações hemodinâmicas provocadas pela arritmia, diferente de pacientes com FA recorrente, melhor adaptados. Além disso, por se tratar de estudo retrospectivo baseado em coleta de dados de prontuários eletrônicos e, também, por não dispor da causa mortis definitiva dos pacientes que evoluíram a óbito, são necessários novos estudos para elucidação dos resultados obtidos.

## Conclusão

No paciente grande idoso (80 anos ou mais) com sepse, o desenvolvimento de FA se mostrou como fator de risco independente para mortalidade intra-hospitalar. Devido às evidências, é cada vez mais urgente abordar este tema nesta população em que a FA incide e tem seu maior impacto. Estes talvez sejam os mais beneficiados ao se manterem no ritmo sinusal ou terem a FA revertida o mais breve possível, visto que são habitualmente mais frágeis e têm menor reserva funcional. Além disso, a identificação de fatores de risco associados à FA no contexto crítico pode servir para eventuais estratégias de controle para prevenção.
