# Supplementary material for: Fibrilação Atrial e Sepse em Pacientes Idosos e sua Associação com Mortalidade Intra-hospitalar
Source: Arq Bras Cardiol. 2023 Feb 23;120(3):e20220295. [Article in Portuguese] doi: 10.36660/abc.20220295 (PMC9972940; doi:10.36660/abc.20220295)
Supplement: Translation

# Translation: Atrial Fibrillation and Sepsis in Elderly Patients and Their Associaton with In-Hospital Mortality

**Authors:** Michele Ouriques Honorato, Juscelio Trajano de Sousa, Luiz Frederico Bezerra Honorato, Nathalia Watanabe, Gabriela Machado Goulart, Rogério Ruscitto do Prado

**Affiliations:** 1 Brasil Hospital Sírio-libanês, São Paulo, SP – Brasil; 2 Brasil Universidade do Extremo Sul Catarinense Curso de Medicina, Criciúma, SC – Brasil; 3 Brasil Hospital Israelita Albert Einstein, São Paulo, SP – Brasil

**Keywords:** Arrhythmias, Cardiac, Atrial Fibrillation, Sepsis, Hospitalization, Hospital Mortality

## Abstract

**Background:**

Atrial fibrillation (AF) affects about 2% to 4% of the world population, and in patients hospitalized in intensive care units, this incidence can reach up to 23% in those with septic shock. The impact of AF in patients with sepsis is reflected in worse clinical outcomes, and the identification of the triggering factors can be a target for future prevention and treatment strategies.

**Objectives:**

To verify the relationship between the development of AF and mortality in patients over 80 years of age included in the sepsis protocol and to identify the risk factors that contribute to the development of AF in this population.

**Methods:**

Retrospective observational study, with a review of electronic medical records and inclusion of 895 patients aged 80 years or older, included in the sepsis protocol of a high-complexity private hospital in São Paulo, SP, from January 2018 to December 2020. All tests were performed with a significance level of 5%.

**Results:**

The incidence of AF in the sample was 13%. After multivariate analysis, using multiple logistic regression, it was possible to demonstrate an association of mortality, in the studied population, with the SOFA score (odds ratio [OR] 1.21 [1.09 – 1.35]), higher values of C-reactive protein (OR 1.04 [1.01 – 1.06]), need for vasoactive drugs (OR 2.4 [1.38 – 4.18]), use of mechanical ventilation (OR 3.49 [1.82 – 6.71]), and mainly AF (OR 3.7 [2.16 – 6.31])

**Conclusion:**

In very elderly patients (80 years of age and older) with sepsis, the development of AF was shown to be an independent risk factor for in-hospital mortality.

## Introduction

Atrial fibrillation (AF) is the most common sustained cardiac arrhythmia, affecting approximately 2% to 4% of the world population, and it implies high morbidity and mortality, as well as high costs for health services.^1 , 2^ Among patients requiring hospitalization for any other reason, AF remains the most detected cardiac arrhythmia, especially in critically ill patients. The risk of developing AF in patients admitted to the intensive care unit ranges from 4.5% to 11%, reaching up to 23% in those with septic shock.^3 , 4^

Among the several established risk factors for AF, age is perhaps the most prominent, and the increase in population longevity is expected to produce an increasing number of new cases,^1 , 2^ which makes the elderly, especially the very elderly (those over 80 years of age), more susceptible to the deleterious effects and already known risks arising from the disease, aggravated further by other comorbidities, which are frequent in this population, and their potential fragility.

Despite the existence of an already well-established relationship between age and AF,^5^ the mechanisms responsible for the development of arrhythmia in critical patients are not yet fully understood. They probably result from accelerated atrial remodeling in combination with triggering factors for arrhythmogenesis, usually found in more severely ill patients, such as inflammation, hydroelectrolytic disorders, and pro-arrhythmic medications, including vasopressors and inotropes.^6^

The impact of AF in this population is reflected by worse outcomes, highlighting for its importance increased hospital stay, which also has an impact on costs, increased mechanical ventilation time and its consequences, and higher mortality.^7 - 10^ However, its importance in the critical environment is still not completely clear, sometimes functioning as a determinant of patients’ worsening, sometimes as a marker of the severity of underlying diseases, and it can even be used as a prognostic factor.^3 , 6^

Within the various uncertainties in the critical environment related to AF, the relationship between arrhythmia and sepsis, especially in the elderly, still raises several theories. Although studies on sepsis have increased in the last decade, few studies have addressed its relationship with AF in a very elderly population (80 years and over). These patients may benefit the most from maintaining sinus rhythm or having AF reversed as soon as possible, as they are usually more fragile and have less functional reserve.

For these reasons, it is important and extremely relevant to conduct studies that address the topic, bringing additional information that can contribute to the current literature. Based on this, the primary objective of this study was to verify the association of in-hospital mortality with the development of AF in patients with sepsis and, as a secondary objective, to identify potential risk factors that contribute to the development of AF in this population and compare the duration of hospitalization between patients who developed AF and those who remained in sinus rhythm.

## Methods

This is a retrospective observational study, with secondary data collection from reviewed electronic medical records of patients aged 80 years or older, included in the sepsis protocol of a high complexity private hospital in São Paulo, SP, from January 2018 to December 2020. The study was approved by the institutional Research Ethics Committee by Hospital Sírio Libanês, under CAAE protocol 47665721.9.0000.546.

To define AF, cardiac rhythm data described in medical records and, when available, 12-lead electrocardiogram recordings were used.

The sepsis protocol used at the institute, which was based on the definition of the 2016 Surviving Sepsis Campaign Guidelines (SEPSIS-3),^11^ consisted of possible or probable focus of infection associated with two of the following systemic inflammatory response syndrome markers: heart rate > 90 bpm, body temperature > 38°C or < 36°C, respiratory rate > 20 bpm, leukocytes > 12.000/mm^3^, or < 4000/mm^3^; or at least one marker of organ dysfunction, characterized by: lactate > 22 mg/dL, creatinine > 2.0 mg/dL, bilirubin > 2.0 mg/dL, international normalization index > 1.5, activated thromboplastin time > 60 seconds, or platelets < 100.000/mm^3^.

A total of 1339 medical records were eligible for inclusion, and 444 who had arrhythmia at admission or who had a cardiac pacemaker were excluded. There remained 895 patients admitted in sinus rhythm, among which 14.9% had previously presented paroxysmal AF ( Figure 1 ).

**Central Illustration:** : Atrial Fibrillation and Sepsis in Elderly Patients and Their Associaton with In-Hospital Mortality

Summary of the main results. AF: atrial fibrillation; CI: confidence interval; OR: odds ratio

The following variables were analyzed: sex, age, body mass index, length of hospital stay, in-hospital mortality, associated comorbidities (systemic arterial hypertension, heart failure [HF], diabetes mellitus, stroke, chronic coronary arterial disease, previous AF, chronic kidney disease, obesity, or others [all comorbidities not mentioned]), previous use of antiarrhythmics, echocardiographic data such as left atrial size and left ventricular ejection fraction (LVEF), focus of sepsis, SOFA score (characterized by the sum of values assigned from 0 to 4 for each of the following variables: PaO_2_/FiO_2_ratio, platelet count, total bilirubin values, mean arterial pressure, Glasgow coma scale, creatinine levels or urine output),^11^ C-reactive protein (CRP) value at admission, use of vasoactive drugs (noradrenaline, vasopressin, and/or dobutamine), and need for mechanical ventilation.

The definition of HF was based on previous comorbidities described in medical records and/or on previous echocardiography exams of the patient demonstrating LVEF <40% (Simpson or Teicholz), which, according to the Brazilian Guideline on Chronic and Acute Heart Failure, characterizes it as HF with reduced ejection fraction.^12^ Enlarged left atrium was defined as linear measurement > 40 mm (reference value between 28 and 40 mm). CRP results above 1 mg/dL, obtained by the ultrasensitive immunoturbidimetry method, should be interpreted as indicative of a possible infectious or inflammatory process.

### Statistical analysis

Qualitative characteristics were described using absolute and relative frequencies, based on the development of AF, and the association of characteristics with groups was verified using chi-square or exact tests (Fisher’s exact test or likelihood ratio test).^13^ Quantitative characteristics were described, according to the development of AF, using mean and standard deviation when data distribution was normal, or median and quartiles when data distribution was not normal. Normality was evaluated using the Kolmogorov-Smirnov test and compared using Student’s t-test (unpaired) or Mann-Whitney tests, respectively.

The unadjusted odds ratios (OR) were estimated for each evaluated characteristic for the outcome using bivariate logistic regression, and the multiple logistic regression model was estimated, selecting the variables that in the bivariate tests presented significance levels below 0,20 (p < 0.20), with all variables inserted in the model kept in the final model (full model).

The analyses were performed using IBM-SPSS for Windows version 22.0 and tabulated using Microsoft-Excel 2010, and the tests were performed with a significance level of 5%.

## Results

The mean age of the population studied was 88.3 years (± 5.3), and 52.4% were women. During hospitalization, 118 patients developed AF, representing approximately 13% of the total number of individuals followed up retrospectively ( Figure 1 ).

As expected, patients who developed AF had a higher frequency of heart failure and previous AF in their history, as well as enlarged left atrium and reduced LVEF on echocardiogram.

Among patients who developed AF, higher SOFA score values and higher levels of CRP were observed.

These patients with arrhythmia remained hospitalized longer, and they required vasoactive drugs and mechanical ventilation more frequently. These data are detailed in Table 1 .

**Table 1:** – Description of the characteristics evaluated according to the development of atrial fibrillation and the result of the statistical tests

Variable	In-hospital atrial fibrillation		Total (N = 895)	p
	No (N = 777)	Yes (N = 118)		
**Age, mean ± SD**	88.2 ± 5.2	89.1 ± 5.6	88.3 ± 5.3	0.080**
**Sex, n (%)**				0.818
Male	371 (47.7)	55 (46.6)	426 (47.6)	
Female	406 (52.3)	63 (53.4)	469 (52.4)	
**Body mass index (admission), mean ± SD**	25.3 ± 4.9	25.1 ± 4.8	25.3 ± 4.9	0.634**
**Length of stay (days), median (IQR)**	10 (7; 16)	13.5 (8; 25.3)	10 (7; 17)	<0.001£
**Systemic arterial hypertension, n (%)**				0.145
No	325 (41.8)	41 (34.7)	366 (40.9)	
Yes	452 (58.2)	77 (65.3)	529 (59.1)	
**Diabetes mellitus, n (%)**				0.42
No	523 (67.3)	75 (63.6)	598 (66.8)	
Yes	254 (32.7)	43 (36.4)	297 (33.2)	
**Heart failure, n (%)**				0.002
No	634 (81.6)	82 (69.5)	716 (80)	
Yes	143 (18.4)	36 (30.5)	179 (20)	
**Stroke, n (%)**				0.321
No	665 (85.6)	105 (89)	770 (86)	
Yes	112 (14.4)	13 (11)	125 (14)	
**Chronic coronary arterial disease, n (%)**				0.647
No	588 (75.7)	87 (73.7)	675 (75.4)	
Yes	189 (24.3)	31 (26.3)	220 (24.6)	
**Atrial fibrillation, n (%)**				<0.001
No	688 (88.5)	74 (62.7)	762 (85.1)	
Yes	89 (11.5)	44 (37.3)	133 (14.9)	
**Non-dialysis chronic kidney disease, n (%)**				0.739*
No	760 (97.8)	115 (97.5)	875 (97.8)	
Yes	17 (2.2)	3 (2.5)	20 (2.2)	
**Obesity, n (%)**				0.969
No	515 (66.3)	78 (66.1)	593 (66.3)	
Yes	262 (33.7)	40 (33.9)	302 (33.7)	
**Others, n (%)** ^**a**^				0.269*
No	24 (3.1)	6 (5.1)	30 (3.4)	
Yes	753 (96.9)	112 (94.9)	865 (96.6)	
**Focus of sepsis, n (%)**				0.886#
Pulmonary	436 (56.1)	71 (60.2)	507 (56.6)	
Urinary	181 (23.3)	23 (19.5)	204 (22.8)	
Abdominal	90 (11.6)	13 (11)	103 (11.5)	
Cutaneous	23 (3)	3 (2.5)	26 (2.9)	
Other^b^	47 (6)	8 (6.8)	55 (6.1)	
**Antiarrhythmic, n (%)**				0.053
No	493 (63.4)	63 (53.4)	556 (62.1)	
Amiodarone	91 (11.7)	24 (20.3)	115 (12.8)	
Beta bloker	149 (19.2)	24 (20.3)	173 (19.3)	
Other^c^	44 (5.7)	7 (5.9)	51 (5.7)	
**Left atrium size (> 40 mm), n (%) &**				0.021
Normal	308 (46.7)	38 (34.9)	346 (45.1)	
Increased	351 (53.3)	71 (65.1)	422 (54.9)	
**Left ventricular ejection fraction (< 40%), n (%) &**				0.04
Normal	621 (94.2)	97 (89)	718 (93.5)	
Decreased	38 (5.8)	12 (11)	50 (6.5)	
**SOFA score on admission, mean ± SD**	3.2 ± 2.1	3.8 ± 2.6	3.3 ± 2.2	0.004**
**C-reactive protein, median (IQR)**	4.6 (1.4; 10.7)	7.6 (2.2; 14)	4.8 (1.4; 11.2)	0.027£
**Vasoactive drugs, n (%)**				<0.001
No	615 (79.2)	65 (55.1)	680 (76)	
Yes	162 (20.8)	53 (44.9)	215 (24)	
**Mechanical ventilation, n (%)**				<0.001
No	729 (93.8)	90 (76.3)	819 (91.5)	
Yes	48 (6.2)	28 (23.7)	76 (8.5)	

Chi-square test; * Fisher's exact test; # Likelihood ratio test; ** Student t-test; £ Mann-Whitney Test; & Not all patients have the information. IQR: interquartile range; SD: standard deviation. a Comorbidities not mentioned. b Catheter, central nervous system, etc. c Calcium channel Blocker, digoxin, propafenone, etc.

The in-hospital mortality rate among patients who developed AF was 34.1% ( Figure 1 ). These patients remained hospitalized longer, and, according to statistical analysis, the following contributed to the unfavorable outcome: comorbidities such as previous HF, the initial focus of sepsis, higher SOFA score, higher CRP values, need for vasoactive drugs, need for mechanical ventilation and, as suspected, the development of AF. These data are detailed in Table 2 .

**Table 2:** – Description of patient outcomes according to the characteristics evaluated and results of unadjusted analyses

Variable	Patient's vital status at discharge		OR	CI (95%)		p
	Alive (N = 766)	Death (N = 129)		Low	High	
**Age, mean ± SD**	88.3 ± 5.2	88.4 ± 5.6	1.00	0.97	1.04	0.815**
**Sex, n (%)**						0.909
Male	364 (85.4)	62 (14.6)	1.00			
Female	402 (85.7)	67 (14.3)	0.98	0.67	1.42	
**Body mass index (admission), mean ± SD**	25.4 ± 4.8	24.8 ± 5.4	0.98	0.94	1.02	0.224**
Length of stay (days), median (IQR)	10 (7; 16)	13 (5; 29)	1.01	1.01	1.02	0.027£
**Systemic arterial hypertension, n (%)**						0.664
No	311 (85)	55 (15)	1.00			
Yes	455 (86)	74 (14)	0.92	0.63	1.34	
**Diabetes mellitus, n (%)**						0.870
No	511 (85.5)	87 (14.5)	1.00			
Yes	255 (85.9)	42 (14.1)	0.97	0.65	1.44	
**Heart failure, n (%)**						0.008
No	624 (87.2)	92 (12.8)	1.00			
Yes	142 (79.3)	37 (20.7)	1.77	1.16	2.70	
**Stroke, n (%)**						0.580
No	657 (85.3)	113 (14.7)	1.00			
Yes	109 (87.2)	16 (12.8)	0.85	0.49	1.50	
**Chronic coronary arterial disease n (%)**						0.298
No	573 (84.9)	102 (15.1)	1.00			
Yes	193 (87.7)	27 (12.3)	0.79	0.50	1.24	
**Atrial fibrillation, n (%)**						0.964
No	652 (85.6)	110 (14.4)	1.00			
Yes	114 (85.7)	19 (14.3)	0.99	0.58	1.67	
**Non-dialysis chronic kidney disease, n (%)**						>0.999*
No	749 (85.6)	126 (14.4)	1.00			
Yes	17 (85)	3 (15)	1.05	0.30	3.63	
**Obesity, n (%)**						0.915
No	507 (85.5)	86 (14.5)	1.00			
Yes	259 (85.8)	43 (14.2)	0.98	0.66	1.45	
**Others, n (%)** ^**a**^						0.295*
No	28 (93.3)	2 (6.7)	1.00			
Yes	738 (85.3)	127 (14.7)	2.41	0.57	10.24	
**Focus of sepsis, n (%)**						0.008#
Pulmonary	442 (87.2)	65 (12.8)	1.00			
Urinary	182 (89.2)	22 (10.8)	0.82	0.49	1.37	
Abdominal	78 (75.7)	25 (24.3)	2.18	1.30	3.67	
Cutaneous	22 (84.6)	4 (15.4)	1.24	0.41	3.70	
Other^b^	42 (76.4)	13 (23.6)	2.11	1.07	4.13	
**Antiarrhythmic, n (%)**						0.889
No	479 (86.2)	77 (13.8)	1.00			
Amiodarone	96 (83.5)	19 (16.5)	1.23	0.71	2.13	
Beta bloker	147 (85)	26 (15)	1.10	0.68	1.78	
Other^c^	44 (86.3)	7 (13.7)	0.99	0.43	2.28	
**Left atrium size (> 40 mm), n (%) &**						0.765
Normal	295 (85.3)	51 (14.7)	1.00			
Increased	363 (86)	59 (14)	0.94	0.63	1.41	
**Left ventricular ejection fraction (< 40%), n (%) &**						0.015
Normal	621 (86.5)	97 (13.5)	1.00			
Decreased	37 (74)	13 (26)	2.25	1.15	4.38	
**SOFA score on admission, mean ± SD**	3 ± 1.9	4.8 ± 2.9	1.40	1.29	1.52	<0.001**
**C-reactive protein, median (IQR)**	4.4 (1.3; 10.6)	8.1 (2.6; 17.9)	1.05	1.03	1.07	<0.001£
**Vasoactive drugs, n (%)**						<0.001
No	626 (92.1)	54 (7.9)	1.00			
Yes	140 (65.1)	75 (34.9)	6.21	4.18	9.22	
**Mechanical ventilation, n (%)**						<0.001
No	731 (89.3)	88 (10.7)	1.00			
Yes	35 (46.1)	41 (53.9)	9.73	5.89	16.08	
**In-hospital atrial fibrillation, n (%)**						<0.001
No	692 (89.1)	85 (10.9)	1.00			
Yes	74 (62.7)	44 (37.3)	4.84	3.13	7.49	

Chi-square test; * Fisher’s exact test; # Likelihood ratio test; ** Student t-test; £ Mann-Whitney Test. CI: confidence interval; IQR: interquartile range; OR: odds ratio; SD: standard deviation. a Comorbidities not mentioned. b Catheter, central nervous system, etc. c Calcium channel blocker, digoxin, propafenone, etc.

After multivariate analysis, using multiple logistic regression, it was possible to demonstrate an association of mortality, in the studied population, with the SOFA score (OR 1.21 [1.09 – 1.35]), higher values of CRP (OR 1.04 [1.01 – 1.06]), need for vasoactive drugs (OR 2.4 [1.38 – 4.18]), use of mechanical ventilation (OR 3.49 [1.82 – 6.71]), and mainly AF (OR 3.7 [2.16 – 6.31]) ( Table 3 ) ( Figure1 ).

**Table 3:** – Result of the model adjusted to explain death in patients of the sepsis protocol aged 80 years or older

Variable	OR	CI (95%)		p

		Low	high	
**Heart failure**	1.60	0.91	2.81	0.104
**Focus of sepsis**				
Pulmonary	1.00			
Urinary	1.26	0.68	2.34	0.457
Abdominal	1.50	0.74	3.05	0.263
Cutaneous	0.78	0.16	3.75	0.752
Other^a^	1.37	0.56	3.35	0.494
Left ventricular eject fraction	1.09	0.47	2.55	0.844
SOFA score on admission	1.21	1.09	1.35	<0.001
**C-reactive protein**	1.04	1.01	1.06	0.007
**Vasoactive drugs**	2.40	1.38	4.18	0.002
**Mechanical ventilation**	3.49	1.82	6.71	<0.001
**In-hospital atrial fibrillation**	3.70	2.16	6.31	<0.001

*Multiple logistic regression (full model). CI: confidence interval; IOR: odds ratio. a Catheter, central nervous system, etc.*

## Discussion

The overall incidence of new or recurrent AF found in our population during hospitalization was 13.2%. The literature demonstrates a great variability of results; for instance, in a meta-analysis conducted by Kuipers et al.,^14^ the incidence of AF in patients with sepsis, severe sepsis, and septic shock was respectively 8%, 10%, and 23%.^15^ Walkey et al.,^17^ including more than 40,000 patients, but only with severe sepsis (old classification), found an incidence of 5.9% for new AF. On the other hand, Meierhenrich et al.,^9^ separating only patients with septic shock, found an incidence of 46% for new AF, 10 times more than patients with sepsis without evolution to shock. However, more recently, the meta-analysis conducted by Corica et al.^18^ showed a 13.5% prevalence of new AF in patients with sepsis, similar to that of the present study. This variability can be explained by the numerous inclusion criteria and different populations addressed. This study, in turn, represents a very specific portion of the elderly population whose incidence of AF is higher, in addition to not differentiating between patients with sepsis and septic shock.

Among the risk factors that contributed to the development of AF during hospitalization, the previous history of HF and AF and echocardiographic findings that characterized left atrial enlargement and LVEF reduction stood out. Several studies that addressed the topic also had similar findings,^4 , 6 , 15^ but there are controversies. Salman et al.,^19^ analyzing a prospective cohort, did not demonstrate a relationship between the presentation of AF and the size of the left atrium, despite associating a decrease in LVEF with a greater chance of progression to arrhythmia. Shaver et al.,^15^ in contrast, managed to demonstrate an association with left atrial size, but not with LVEF. Although it was not the subject of this work, laboratory tests with brain natriuretic peptide place it as an independent marker in the development of AF, as mentioned by Augusto et al.^20^ This once again confirms that HF is a predictor for the onset of arrhythmia, both on an outpatient basis and in a critical setting.^21 - 23^

In this study, among patients who developed AF, higher SOFA score values and higher CRP levels were also observed. Currently, the inflammatory role generated by sepsis is significant in the development and maintenance of AF. Steinber et al.^24^ highlight that the inflammatory process predisposes to oxidative stress, apoptosis, and fibrosis, generating an important substrate to trigger arrhythmia. Another aggravating factor in this context is the prothrombotic environment produced by inflammation, capable of inducing endothelial dysfunction, platelet activation, and the coagulation cascade. Both effects may be responsible not only for the initiation and maintenance of AF, but also for worsening thrombotic outcomes associated with arrhythmia.^24^ These findings were reinforced by Harada et al.,^25^ who also associated higher CRP and SOFA scores with the development of AF, including mortality, after adjusted analysis. Launey et al.^23^ also found higher SOFA scores in patients who developed AF. Meierhenrich et al.^9^ showed that patients who developed AF, whether septic or not, had higher levels of CRP before the arrhythmic event. Chung et al.^26^ also demonstrated an association between high CRP values and the occurrence and maintenance of AF. In these cases, elevated CRP points to an inflammatory state that promotes the development or persistence of AF.

Initially, sepsis from an abdominal or other focus (non-urinary, pulmonary, or cutaneous) was associated with higher mortality; however, in the multivariate analysis, no statistical difference was found. Although there are data indicating a higher incidence of AF with respiratory tract infections and urinary focus,^10 , 14 , 16 , 23^ in our study, it was not possible to determine the relationship between the sites of infection and the incidence of AF.

In addition to longer hospital stay in this study, patients who developed AF also required vasoactive drugs and mechanical ventilation more often. The use of mechanical ventilation has already been evidenced as a risk factor for AF in several studies,^4 , 7 , 21^ but there is still no consensus.^15^ In patients with sepsis who progress to shock, vasoactive drugs become imperative due to the failure to compensate between the demand and supply of oxygen to the tissues; therefore, it is already a factor of worse prognosis. This instability can result in AF, just as the arrhythmia itself generates the need for higher doses of vasopressors. This relationship has already been reproduced in some studies.^4 , 15 , 22^

Several previous studies failed to find a direct association of new or recurrent AF with the outcome of death during hospitalization, but the results found here suggest that the development of AF in septic patients in this age group has a strong impact on in-hospital mortality. This finding is in agreement with other recent studies, including meta-analyses of critically ill patients with sepsis and/or septic shock who developed new AF, which showed similar results.^8 , 10 , 13 , 14^

There has always been great discussion as to whether AF plays a role in the course or outcome of sepsis, or simply reflects the severity of the disease, as a marker of severity. As in other previously mentioned studies, in this study, AF did represent an organic dysfunction that implied a worsening of the clinical outcome. Given the above, the appropriate approach to arrhythmia must be raised to another level of importance in the context of the evolution of this group of patients, requiring the development of adequate prevention and treatment strategies, with the aim of reducing health damage.^15^ Specifically on this topic, there are already results showing that failure to maintain sinus rhythm in patients with sepsis was associated with worse mortality rates compared to patients whose AF was successfully reversed.^9 , 16^

This study has some limitations, such as the definition of AF, which was based on clinical records and, when available, 12-lead electrocardiogram. This leaves room for possible episodes of paroxysmal AF not diagnosed or recorded by the attending physician. Patients with recurrent AF (37.3%) were also included, but this was not shown to be a sampling bias with implications for outcomes. This fact had already been addressed in studies carried out by Arrigo et al.^3^ and Shaver et al.,^15^ showing higher mortality rates in patients with new AF, probably due to a lower tolerance of hemodynamic changes caused by arrhythmia, unlike patients with recurrent AF, who are better adapted. In addition, as this is a retrospective study based on data collection from electronic medical records and because the definitive causes of death of patients are unknown, further studies are needed to elucidate the results obtained.

## Conclusion

In very elderly patients (80 years and over) with sepsis, the development of AF was shown to be an independent risk factor for in-hospital mortality. Due to the evidence, it is increasingly urgent to address this issue in this population, where AF has the greatest impact. These patients may benefit the most from maintaining sinus rhythm or having AF reversed as soon as possible, since they are usually more fragile and have less functional reserve. In addition, the identification of risk factors associated with AF in the critical context can serve for eventual control strategies for prevention.
